# Exposure to secondhand smoke from cigarettes and secondhand aerosol from tobacco and nicotine products in indoor and outdoor public spaces in the European Union: a cross-sectional study

**DOI:** 10.1136/bmjph-2025-002903

**Published:** 2025-07-31

**Authors:** Charlotte Xin Li, Pin-Chun Wang, Ariadna Feliu, Anthony A Laverty, Cristina Martinez, Armando Peruga, Charis Girvalaki, Cornel Radu Loghin, Constantine I Vardavas, Filippos T Filippidis

**Affiliations:** 1 Department of Primary Care and Public Health, Imperial College London, London, UK; 2 European Network for Smoking and Tobacco Prevention, Brussels, Belgium; 3 CIBER en Enfermedades Respiratorias (CIBERES), Madrid, Spain; 4 Tobacco Control Unit, Cancer Control and Prevention Program, Institut Catala d’Oncologia, L’Hospitalet de Llobregat, Spain; 5 Cancer Control and Prevention Group, Institut d’Investigació Biomèdica de Bellvitge-IDIBELL, L’Hospitalet de Llobregat, Spain; 6 Department of Public Health, Maternal Health and Mental Health, School of Nursing, Universitat de Barcelona, Barcelona, Spain; 7 Philip R. Lee Institute for Health Policy Studies, University of California San Francisco, San Francisco, California, USA; 8 Laboratory of Epidemiology, Hygiene and Medical Statistics, School of Medicine, National and Kapodistrian University of Athens, Athens, Greece

**Keywords:** Public Health, Environmental Exposure, Preventive Medicine

## Abstract

**Introduction:**

In December 2024, the Council of the European Union (EU) adopted a recommendation to expand regulations on the use of nicotine-containing products both in indoor settings and specific outdoor areas. This study aimed to examine sociodemographic factors associated with exposure to conventional tobacco smoke and aerosols across the EU and support for relevant regulations.

**Methods:**

We performed a secondary analysis of cross-sectional data from the Special Eurobarometer 99.3 (n=26 358, May–June 2023) across 27 EU Member States (MS). We estimated the weighted prevalence of secondhand exposure to tobacco smoke and aerosols and support for bans on smoking, e-cigarettes and heated tobacco products in public settings. Multilevel Poisson regression models explored associations between sociodemographic factors and these outcomes.

**Results:**

Exposure to secondhand smoke and aerosols varied across EU MS. Younger individuals, those with higher education, living with children, and current and former tobacco and nicotine users (prevalence ratio (PR) 1.16, 1.01–1.33; and PR 1.22, 1.05–1.41, respectively) were more likely to report exposure to both tobacco smoke and aerosol from emerging products. Women (PR 1.05, 1.02–1.08; and PR 1.03, 1.02–1.05, respectively), those living with children (PR 1.05, 1.02–1.07; and PR 1.04, 1.02–1.07, respectively) and those with higher education levels (PR 1.10, 1.04–1.15; and PR 1.10, 1.06–1.14) were more likely to support bans, whereas those with financial difficulties (PR 0.94, 0.89–0.99 and PR: 0.95, 0.91–0.99, respectively), as well as current and former smokers (PR 0.61, 0.55–0.67; and PR 0.78, 0.73–0.84, respectively) and emerging product users (PR 0.84, 0.76–0.92; and PR 0.69, 0.62–0.76, respectively), were less supportive.

**Conclusion:**

Our analysis found that both exposure to secondhand smoke and aerosol and support for bans in public spaces vary substantially between population subgroups and across countries. Our findings can support EU MS in implementing targeted interventions to increase population support for and implement the recent EU Council recommendations.

**WHAT IS ALREADY KNOWN ON THIS TOPIC**

- Public support for smoke-free environments in indoor settings is high in the European Union (EU), but there are inequities in exposure to secondhand smoke. Despite this, little is known about exposure to secondhand smoke from combustible tobacco and secondhand aerosol from e-cigarettes and heated tobacco products in outdoor settings across the EU. Additionally, there is a lack of extensive research on public support for smoke-free and aerosol-free policies in outdoor spaces in the EU.

**WHAT THIS STUDY ADDS**

- This study reveals significant variation in outdoor secondhand smoke and aerosol exposure across the EU, with sociodemographic factors and tobacco and nicotine use influencing exposure levels. Public support for smoke-free and aerosol-free policies also varies by country and socioeconomic status, though the majority of respondents support more comprehensive and stricter smoke- and aerosol-free policies.

**HOW THIS STUDY MIGHT AFFECT RESEARCH, PRACTICE OR POLICY**

- Following the EU’s recent Council Recommendation on smoke- and aerosol-free environments, our findings emphasise the need for more targeted policies, particularly to protect vulnerable groups. With governments now responsible for implementing smoke- and aerosol-free legislation, our study provides crucial evidence to encourage such actions across the EU.

## Introduction

 Tobacco remains one of the most preventable health threats in the European Union (EU), responsible for nearly 700 000 deaths annually.^1^ All forms of tobacco use are harmful, with no safe level of exposure.^2 3^ This includes not only the most prevalent form, cigarette smoking, but also emerging products such as e-cigarettes and heated tobacco products (HTPs), which have been growing in popularity.^4^ Although these products are often perceived as less harmful than traditional combustible cigarettes, they still pose significant health risks.^5^ Besides endangering individual health of those who smoke, tobacco combustion produces secondhand smoke (SHS) that increases the risk of lung cancer, coronary heart disease and other conditions in non-smokers.^6^ While emerging products such as e-cigarettes and HTPs do not produce smoke but rather an aerosol, there are concerns about their potential adverse health effects. E-cigarette and HTP aerosols are composed of complex mixtures of harmful substances, including nicotine, propylene glycol, vegetable glycerin and volatile organic compounds.^7^ Although the long-term effects of secondhand aerosol (SHA) exposure remain under investigation, SHA exposure appears to negatively affect adolescents’ respiratory health.^8^

Exposure to SHS is periodically measured by Eurobarometer surveys across the EU, with recent data indicating notable variation in exposure levels.^9^ Other studies support this finding, with one revealing that Hungary, Romania and Spain have stronger indoor smoking bans and more comprehensive tobacco control policies, resulting in lower levels of exposure compared with Germany and Poland.^10 11^ It is also well-established that the burden of SHS exposure disproportionately affects vulnerable populations, including children, adolescents and those with financial difficulties, exacerbating health disparities across the EU.^12^,^16^ The few existing studies on SHA exposure in indoor public spaces indicate that younger individuals, those with higher education and current or former e-cigarette users report higher levels of exposure to SHA in the EU.^17^ However, most of the literature focuses on exposure in indoor settings and rarely includes assessment of exposure to SHAl outdoors.

In December 2024, the Council of the European Union adopted a new Council Recommendation on smoke- and aerosol-free environments, which calls on MS to extend their smoke-free policies to key outdoor areas, including public playgrounds, amusement parks, swimming pools and public transport stations, to ensure effective protection of bystanders, particularly children and adolescents. Moreover, the Council Recommendation also advises MS to expand all smoke-free policies to include emerging products.^18^ As of early 2025, many EU MS have enacted smoke-free legislation banning smoking in enclosed public spaces, public transport and workplaces, with limited exceptions.^19^ However, the effectiveness of these regulations varies considerably due to inconsistent enforcement at the national and regional levels. Complex legislation, particularly when exemptions are included, has proven challenging to enforce in some EU MS, resulting in compliance issues.^19^ Therefore, to ensure stronger protection of bystanders, particularly of vulnerable populations, comprehensive smoke-free regulations with few exemptions are recommended.^11^ Overall, successful implementation and enforcement of such policies rely heavily on public support.^20^ Therefore, understanding the factors that shape public support for newly proposed measures is crucial for identifying target groups and tailoring strategies.

Despite growing attention to SHS and SHA, there is a significant gap in understanding outdoor exposure levels across the EU. Furthermore, limited research has examined public support for smoke-free and aerosol-free policies in outdoor spaces, particularly within the EU context. In light of the adoption of the new Council Recommendation on smoke- and aerosol-free environments, we used data from the 27 EU MS collected in 2023 to examine the self-reported exposure to SHS and aerosol in various indoor and outdoor settings and levels of public support for the proposed outdoor smoking ban policies, and to explore sociodemographic variations in these outcomes.

## Methods

### Data source

We performed a secondary analysis using data from all 27 EU MS using Special Eurobarometer 99.3, conducted from 10 May 2023 to 5 June 2023, which included 26 358 respondents aged ≥15 years, residing in each of the EU MS. The Eurobarometer survey employed a multistage sampling method: households were sampled using standard ‘random route’ procedures, and one respondent was randomly selected based on household size to participate in interviews and provide self-reported data in the relevant national language.^9^ Consent for participation was requested at the beginning of each interview. To ensure the samples were nationally representative, the datasets were weighted to account for age, sex and area of residence.

### Measures

#### Exposure to secondhand smoke from conventional tobacco products

Participants were asked if, in the last 6 months, people were smoking in (1) indoor public spaces where people normally do not smoke (eg, restaurants, bars, shopping malls, airports, concert halls), (2) an outdoor terrace of a drinking or eating establishment, (3) outdoor spaces intended for use by children or adolescents (eg, nursery and school courtyards, playgrounds), (4) outdoor events (eg, open-air concerts, sports matches, markets), (5) outdoor public spaces (eg, parks, beaches, entrances to public buildings) and (6) open-air public transport stations (eg, bus, tram or train stations). The percentage of ‘yes’ responses over the total number of valid responses (‘yes’, ‘no’, ‘I have not visited in the last 6 months’, and ‘don’t know’) was considered a proxy of the prevalence of exposure to SHS.

#### Exposure to secondhand aerosol from emerging tobacco products

Participants were similarly asked if they had observed the use of e-cigarettes or HTPs in the same six locations over the past 6 months, with the same response options as above, and the percentage of ‘yes’ responses was considered as a proxy for exposure to SHA.

#### Support for tobacco control policies

All participants were asked, ‘Would you be in favour or not in favour of any of the following measures? (1) Banning of smoking in outdoor places where social distancing cannot be ensured (eg, parks, beaches, entrances to public buildings); (2) Banning the use of e-cigarettes or HTPs in environments where smoking is prohibited’” Responses were categorised as either in favour or not in favour/don’t know.

#### Covariates

The survey collected self-reported data on gender (male; female). The 57 responses for ‘none of the above’, ‘non-binary’, ‘do not identify as male/female’ or ‘prefer not to say’ were considered missing values due to very low number. Data were also collected on age (15–24; 25–39; 40–54; 55+ years), self-reported community type (rural, ie, ‘rural area or village’; and urban, ie, ‘small or middle-sized town’ or ‘large town’), age at which participants stopped full-time education (0–15; 16–19; 20+; still studying), living with children (no; yes), employment status (employed; unemployed; students/house persons/retired) and difficulties paying bills at the end of the month over the past 12 months (almost never/never; from time to time/most of the time).

Current tobacco smoking status was evaluated using the question, ‘Regarding smoking cigarettes, cigars, cigarillos or a pipe, which of the following applies to you?’. Respondents could select from the following options: never smoker, current smoker or former smoker. The current use of e-cigarettes and HTPs was assessed through two separate questions. The first question asked, ‘Thinking about the following products, which of the following applies to you? E-cigarettes?’ Respondents could choose from the following options: never used, currently using, used to use but have stopped or tried it only once or twice. A similar question was asked about HTPs. The responses to these two questions were recoded and combined into a single variable, which was categorised into three groups to be consistent with the exposure and policy support questions: (1) never used e-cigarettes or HTPs, (2) current user of at least one product and (3) former user of at least one product (used to use but have stopped or tried only once or twice).

### Statistical analysis

We estimated the weighted prevalence of secondhand exposure to traditional and emerging products over the past 6 months in indoor and outdoor public spaces, as well as the prevalence of public support for banning tobacco products in public spaces across each EU MS. Official weights provided in the Eurobarometer dataset were used.^9^ We conducted multilevel Poisson regression analyses for each of the outcome variables, which allowed for clustering of observations within countries. All models were adjusted for gender, age, age at completion of full-time education, difficulties in paying bills, community type (rural/urban), employment status, living with children, current smoking and current use of e-cigarettes and HTPs. We also conducted a sensitivity analysis, where the ‘don’t know’ responses were excluded, to assess the robustness of the findings. All statistical analyses were performed using Stata 17, while maps were generated in R. Observations with missing values were excluded from the analysis. As the Eurobarometer data is open-access and anonymised, ethical approval from an Institutional Review Board was not required for this secondary analysis. Consent for participation was requested at the beginning of each interview.

### Patient and public involvement

There was no involvement of patients or the public in the design, conduct, reporting or dissemination plans of our research.

## Results

### Exposure to secondhand smoke and aerosol

Sample characteristics are shown in online supplemental table 1. In the EU overall, exposure to SHS and secondhand e-cigarette and HTP aerosol varied substantially across settings (online supplemental tables 2 and 3). SHS exposure in indoor public spaces was reported by 21.5% of participants (95% CI 20.7 to 22.4), while exposure to e-cigarettes and HTPs was 35.8% (34.8–36.7). Substantial variation was observed between countries, with the highest prevalence of exposure reported on outdoor public places and the lowest in indoor public spaces for both traditional and emerging products.

Women were less likely to report conventional tobacco smoke exposure in indoor public spaces (Prevalence Ratio (PR) 0.89; 95% CI 0.84 to 0.94) and at outdoor events (PR: 0.95; 0.92–0.98) compared with men, but there were no gender differences in other settings (table 1). Younger people aged 15–39 years (compared with those 55+) and those still studying or with higher education (compared with those in the lowest education category) were more likely to report exposure to smoke from conventional tobacco products in all settings, although the effect sizes varied. People living with children were more likely to report exposure to tobacco smoke in all outdoor settings, whereas unemployed participants reported higher exposure only in indoor public spaces (PR: 1.10; 1.02–1.20). People not working (students, house persons and retired) were less likely to report exposure to tobacco smoke across all settings assessed.

**Table 1:** Multilevel Poisson regression estimated associations between sociodemographic and behavioural factors and exposure to traditional tobacco smoke in the past 6 months in EU member states (n=25 219)

Variables	In the last 6 months, were people smoking…					
	In indoor public spaces	On outdoor terraces	In outdoor spaces for children or adolescents	At outdoor events	In outdoor public spaces	At open-air public transportation stations
	PR (95% CI)					
Gender						
Male (ref.)	1	1	1	1	1	1
Female	0.89 (0.84 to 0.94)	0.98 (0.96 to 1.00)	1.03 (0.99 to 1.08)	0.95 (0.92 to 0.98)	0.99 (0.97 to 1.01)	1.00 (0.98 to 1.02)
Age (years)						
55+ (ref.)	1	1	1	1	1	1
15–24	1.38 (1.16 to 1.64)	1.11 (1.02 to 1.22)	1.34 (1.17 to 1.53)	1.12 (1.06 to 1.19)	1.11 (1.07 to 1.16)	1.13 (1.07 to 1.20)
25–39	1.15 (1.05 to 1.26)	1.06 (1.01 to 1.11)	1.18 (1.10 to 1.27)	1.12 (1.08 to 1.16)	1.06 (1.03 to 1.09)	1.07 (1.03 to 1.11)
40–54	1.02 (0.96 to 1.09)	1.04 (1.01 to 1.07)	1.03 (0.98 to 1.09)	1.05 (1.03 to 1.08)	1.04 (1.02 to 1.06)	1.04 (1.01 to 1.07)
Difficulty paying bills						
Almost never/never (ref.)	1	1	1	1	1	1
From time to time/most of the time	1.08 (0.98 to 1.20)	0.96 (0.92 to 1.01)	1.03 (0.97 to 1.11)	0.97 (0.92 to 1.01)	0.96 (0.91 to 1.01)	0.98 (0.93 to 1.03)
Community type						
Rural (ref.)	1	1	1	1	1	1
Urban	0.98 (0.86 to 1.12)	1.02 (0.98 to 1.05)	0.99 (0.91 to 1.07)	1.00 (0.97 to 1.03)	1.02 (0.99 to 1.06)	1.03 (0.99 to 1.08)
Education (age at completion)						
0–15 years (ref.)	1	1	1	1	1	1
16–19 years	1.07 (0.94 to 1.21)	1.08 (1.03 to 1.14)	1.03 (0.93 to 1.14)	1.13 (1.07 to 1.20)	1.10 (1.05 to 1.15)	1.11 (1.05 to 1.18)
20+ years	1.08 (0.94 to 1.25)	1.15 (1.08 to 1.21)	1.04 (0.94 to 1.16)	1.17 (1.10 to 1.25)	1.14 (1.07 to 1.20)	1.16 (1.09 to 1.23)
Still studying	1.34 (1.10 to 1.62)	1.22 (1.11 to 1.35)	1.26 (1.10 to 1.45)	1.43 (1.30 to 1.57)	1.24 (1.16 to 1.32)	1.25 (1.15 to 1.35)
Living with children						
No (ref.)	1	1	1	1	1	1
Yes	1.02 (0.95 to 1.11)	1.05 (1.02 to 1.08)	1.12 (1.06 to 1.17)	1.05 (1.03 to 1.08)	1.04 (1.02 to 1.06)	1.03 (1.00 to 1.05)
Employment status						
Employed (ref.)	1	1	1	1	1	1
Unemployed	1.10 (1.02 to 1.20)	0.99 (0.94 to 1.04)	0.99 (0.91 to 1.07)	0.98 (0.93 to 1.04)	1.03 (0.99 to 1.07)	1.04 (1.00 to 1.09)
Students/house persons/retired	0.87 (0.79 to 0.94)	0.93 (0.89 to 0.97)	0.91 (0.86 to 0.97)	0.87 (0.84 to 0.90)	0.92 (0.88 to 0.96)	0.93 (0.90 to 0.97)
Current smoking status						
Never smoker (ref.)	1	1	1	1	1	1
Current smoker	1.06 (0.96 to 1.17)	1.15 (1.10 to 1.20)	1.09 (1.02 to 1.15)	1.13 (1.09 to 1.18)	1.11 (1.07 to 1.14)	1.10 (1.06 to 1.14)
Former smoker	1.04 (0.95 to 1.14)	1.11 (1.08 to 1.14)	1.08 (1.03 to 1.14)	1.11 (1.07 to 1.14)	1.09 (1.07 to 1.12)	1.06 (1.03 to 1.09)
Current status of e-cigarette and HTP use						
Never used e-cigarettes or HTPs (ref.)	1	1	1	1	1	1
Current user of at least one product	1.16 (1.01 to 1.33)	1.03 (0.97 to 1.09)	1.02 (0.93 to 1.12)	1.04 (0.99 to 1.10)	1.01 (0.96 to 1.06)	0.97 (0.92 to 1.03)
Former user of at least one product	1.22 (1.05 to 1.41)	1.05 (1.00 to 1.10)	1.03 (0.94 to 1.12)	1.05 (1.00 to 1.09)	1.04 (1.00 to 1.08)	1.03 (0.99 to 1.06)

Indoor public apaces: indoor public spaces where people normally do not smoke (eg, restaurants, bars, shopping malls, airports, concert halls).

Outdoor terraces: outdoor terraces of a drinking or eating establishment?

Outdoor spaces: outdoor spaces intended for use by children or adolescents (eg, nursery and school courtyard, playgrounds).

Outdoor events: outdoor events (eg, open-air concerts, sport matches, markets).

Outdoor public spaces: outdoor public spaces (eg, park, beach, entrance to public buildings).

Open-air public transportation stations: open-air public transportation stations (eg, bus, tram or train stations).

HTP, heated tobacco product; PR, prevalence ratio.

Current and former smokers were more likely to report tobacco smoke exposure across all outdoor settings compared with never smokers. In contrast, the association between current and former users of emerging products and exposure to conventional tobacco smoke was strongest in indoor public places (PR 1.16; 1.01–1.33; and PR 1.22; 1.05–1.41, respectively).

Younger people (aged 15–39), males, those still studying or with higher education and those living in urban areas were more likely to be exposed to aerosol from e-cigarette or HTP in the majority of indoor and outdoor settings (table 2). Similar to smoke from conventional tobacco products, living with children was associated with higher exposure to SHA in outdoor environments, whereas people not working were less likely to report exposure in all settings assessed. Current and former smokers, as well as current and former users of emerging products, were consistently more likely to report that someone used an e-cigarette or HTP in each of the settings in the past 6 months.

**Table 2:** Multilevel Poisson regression estimated associations between sociodemographic and behavioural factors and exposure to emerging tobacco product aerosol in the past 6 months in EU member states (n=25 219)

Variables	In the last 6 months, were people using e-cigarettes or heated tobacco products…					
	In indoor public spaces	On outdoor terraces	In outdoor spaces for children or adolescents	At outdoor events	In outdoor public spaces	At open-air public transportation stations
	PR (95% CI)					
Gender						
Male (ref.)	1	1	1	1	1	1
Female	0.96 (0.93 to 0.99)	0.98 (0.95 to 1.00)	1.00 (0.97 to 1.04)	0.95 (0.93 to 0.98)	0.98 (0.96 to 1.00)	0.97 (0.95 to 0.99)
Age (years)						
55+ (ref.)	1	1	1	1	1	1
15–24	1.40 (1.25 to 1.56)	1.21 (1.11 to 1.31)	1.36 (1.22 to 1.52)	1.20 (1.12 to 1.29)	1.17 (1.11 to 1.24)	1.18 (1.12 to 1.24)
25–39	1.30 (1.21 to 1.40)	1.12 (1.07 to 1.17)	1.22 (1.15 to 1.29)	1.16 (1.12 to 1.21)	1.10 (1.07 to 1.13)	1.12 (1.09 to 1.16)
40–54	1.15 (1.09 to 1.22)	1.09 (1.06 to 1.12)	1.09 (1.04 to 1.15)	1.11 (1.07 to 1.14)	1.08 (1.05 to 1.11)	1.09 (1.06 to 1.13)
Difficulty paying bills						
Almost never/never (ref.)	1	1	1	1	1	1
From time to time/most of the time	0.97 (0.89 to 1.05)	0.99 (0.95 to 1.03)	1.00 (0.95 to 1.07)	0.99 (0.94 to 1.04)	0.98 (0.93 to 1.02)	0.99 (0.95 to 1.03)
Community type						
Rural (ref.)	1	1	1	1	1	1
Urban	1.04 (0.95 to 1.13)	1.06 (1.02 to 1.10)	1.03 (0.95 to 1.12)	1.05 (1.01 to 1.08)	1.07 (1.04 to 1.11)	1.08 (1.04 to 1.12)
Education (age at completion)						
0–15 years (ref.)	1	1	1	1	1	1
16–19 years	1.18 (1.02 to 1.36)	1.14 (1.06 to 1.22)	1.10 (0.99 to 1.22)	1.20 (1.11 to 1.30)	1.16 (1.08 to 1.24)	1.19 (1.11 to 1.27)
20+ years	1.26 (1.07 to 1.48)	1.21 (1.12 to 1.31)	1.16 (1.04 to 1.30)	1.26 (1.16 to 1.37)	1.22 (1.12 to 1.32)	1.24 (1.15 to 1.33)
Still studying	1.69 (1.37 to 2.08)	1.42 (1.27 to 1.59)	1.46 (1.25 to 1.70)	1.54 (1.36 to 1.76)	1.44 (1.30 to 1.61)	1.43 (1.30 to 1.57)
Living with children						
No (ref.)	1	1	1	1	1	1
Yes	1.04 (0.98 to 1.10)	1.06 (1.03 to 1.10)	1.11 (1.05 to 1.16)	1.05 (1.02 to 1.08)	1.06 (1.03 to 1.09)	1.04 (1.01 to 1.07)
Employment status						
Employed (ref.)	1	1	1	1	1	1
Unemployed	1.07 (0.99 to 1.15)	0.98 (0.93 to 1.03)	1.01 (0.94 to 1.09)	1.00 (0.95 to 1.05)	1.01 (0.96 to 1.05)	1.04 (0.99 to 1.10)
Students/house persons/retired	0.81 (0.74 to 0.88)	0.85 (0.80 to 0.90)	0.84 (0.78 to 0.90)	0.82 (0.78 to 0.87)	0.85 (0.81 to 0.89)	0.87 (0.83 to 0.91)
Current smoking status						
Never smoker (ref.)	1	1	1	1	1	1
Current smoker	1.27 (1.20 to 1.35)	1.17 (1.13 to 1.22)	1.20 (1.13 to 1.26)	1.14 (1.09 to 1.20)	1.14 (1.09 to 1.19)	1.14 (1.09 to 1.19)
Former smoker	1.22 (1.17 to 1.28)	1.16 (1.13 to 1.19)	1.15 (1.10 to 1.21)	1.15 (1.11 to 1.18)	1.13 (1.10 to 1.17)	1.11 (1.08 to 1.15)
Current status of e-cigarette and HTP use						
Never used e-cigarettes or HTPs (ref.)	1	1	1	1	1	1
Current user of at least one product	1.14 (1.03 to 1.26)	1.12 (1.04 to 1.20)	1.09 (0.99 to 1.20)	1.14 (1.08 to 1.20)	1.11 (1.06 to 1.16)	1.09 (1.03 to 1.15)
Former user of at least one product	1.22 (1.14 to 1.30)	1.13 (1.07 to 1.19)	1.13 (1.06 to 1.20)	1.12 (1.07 to 1.17)	1.09 (1.04 to 1.15)	1.09 (1.04 to 1.13)

Indoor public spaces: Indoor public spaces where people normally do not smoke (eg, restaurants, bars, shopping malls, airports, concert halls).

Outdoor terraces: outdoor terraces of a drinking or eating establishment.

Outdoor spaces: outdoor spaces intended for use by children or adolescents (eg, nursery and school courtyard, playgrounds).

Outdoor events: outdoor events (eg, open-air concerts, sport matches, markets).

Outdoor public spaces: outdoor public spaces (eg, park, beach, entrance to public buildings).

Open-air public transportation stations: open-air public transportation stations (eg, bus, tram or train stations).

HTP, heated tobacco product; PR, prevalence ratio.

### Support for tobacco control policies

**Figure 1:**
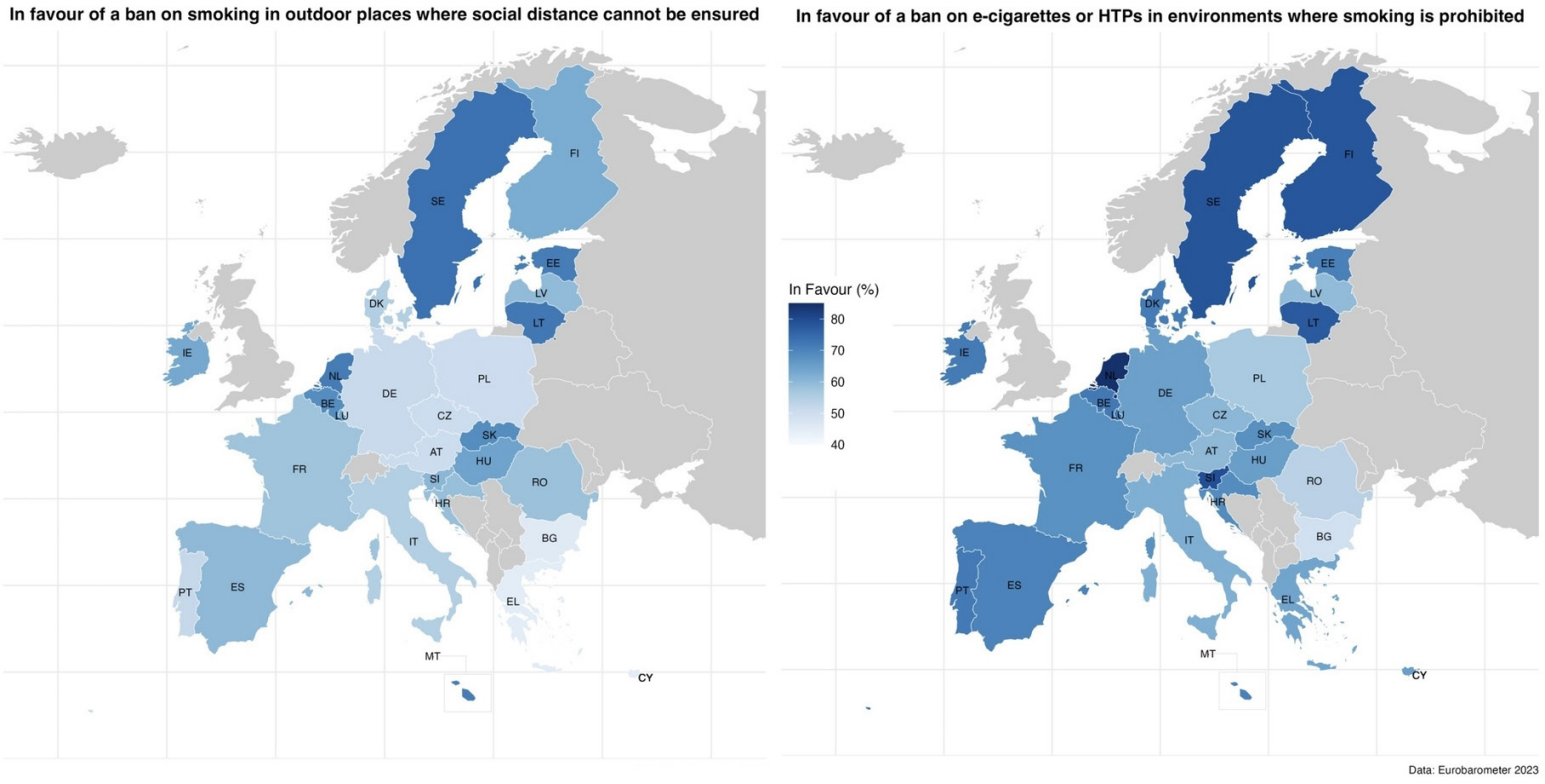
Public support for smoking and e-cigarette/heated tobacco product bans in public spaces across 27 European Union (EU) Member States (n=26 358). HTPs, heated tobacco products.

A total of 56.5% (55.5–57.5) of respondents supported banning smoking in outdoor areas where social distancing cannot be ensured across the EU. Support was highest in Sweden (73.4%; 69.9–76.7) and lowest in Eastern and some Southern European countries, such as Greece (44.9%; 41.8–48.0), Cyprus (44.5%; 39.8–49.3) and Bulgaria (45.6%; 42.5–48.7). Support for banning e-cigarettes and HTPs in environments where smoking is prohibited was 65.8% (64.9–66.8) overall, highest in the Netherlands (84.4%; 81.5–86.9) and lowest in Eastern Europe, with countries like Bulgaria (49.3%; 46.2–52.4) and Romania (53.8%; 50.7–56.9) reporting the least endorsement of the policy (figure 1).

The associations between sociodemographic factors and support for bans were very consistent in the two assessed policies (table 3). Compared with men, women were more likely to support outdoor smoking bans (PR: 1.05; 1.02–1.08) and restrictions on e-cigarettes or HTPs in smoke-free environments (PR: 1.03; 1.02–1.05). Individuals with financial difficulties were less likely to support outdoor smoking bans (PR: 0.94, 0.89–0.99) and e-cigarette or HTP restrictions (PR: 0.95, 0.91–0.99). Those who completed their education at 20+years were 10% more likely to support both outdoor smoking bans (PR: 1.10, 1.04–1.15) and restrictions on e-cigarettes or HTPs (PR: 1.10, 1.06–1.14), compared with those who finished by age 15 or younger, while those living with children were more likely to support both outdoor smoking bans and restrictions on emerging products (PR: 1.05, 1.02–1.07 and PR: 1.04, 1.02–1.07, respectively), than those living without children. Current smokers were 39% and 22% less likely to support outdoor smoking bans (PR: 0.61, 0.55–0.67) and e-cigarette or HTP restrictions (PR: 0.78, 0.73–0.84) relative to non-smokers. Similarly, current e-cigarette or HTP users were 16% less likely to support outdoor smoking bans (PR: 0.84, 0.76–0.92) and 31% less likely to support e-cigarette or HTP restrictions (PR: 0.69, 0.62–0.76) than non-users.

**Table 3:** Multilevel Poisson regression estimating associations between sociodemographic and behavioural factors and attitudes towards banning tobacco products in public spaces in Europen Union (EU) member states (n=25 219)

Variables	In favour of a ban on smoking in outdoor places where social distance cannot be ensured	In favour of a ban on e-cigarettes or HTPs in environments where smoking is prohibited
	PR (95% CI)	
Gender		
Male (ref.)	1	1
Female	1.05 (1.02 to 1.08)	1.03 (1.02 to 1.05)
Age (years)		
55+ (ref.)	1	1
15–24	1.03 (0.97 to 1.10)	1.00 (0.95 to 1.06)
25–39	0.97 (0.93 to 1.01)	0.99 (0.97 to 1.03)
40–54	0.99 (0.96 to 1.02)	1.00 (0.97 to 1.03)
Difficulty paying bills		
Almost never/never (ref.)	1	1
From time to time/most of the time	0.94 (0.89 to 0.99)	0.95 (0.91 to 0.99)
Community type		
Rural (ref.)	1	1
Urban	1.01 (0.97 to 1.06)	1.01 (0.97 to 1.06)
Education (age at completion)		
0–15 years (ref.)	1	1
16–19 years	1.04 (1.00 to 1.08)	1.03 (0.99 to 1.07)
20+ years	1.10 (1.04 to 1.15)	1.10 (1.06 to 1.14)
Still studying	1.00 (0.93 to 1.07)	1.05 (0.99 to 1.12)
Living with children		
No (ref.)	1	1
Yes	1.05 (1.02 to 1.07)	1.04 (1.02 to 1.07)
Employment status		
Employed (ref.)	1	1
Unemployed	1.03 (0.96 to 1.10)	1.02 (0.98 to 1.07)
Students/house persons/retired	1.08 (1.04 to 1.12)	1.04 (1.01 to 1.07)
Current smoking status		
Never smoker (ref.)	1	1
Current smoker	0.61 (0.55 to 0.67)	0.78 (0.73 to 0.84)
Former smoker	0.92 (0.88 to 0.96)	0.99 (0.96 to 1.02)
Current status of e-cigarette and HTP use		
Never used e-cigarettes or HTPs (ref.)	1	1
Current user of at least one product	0.84 (0.76 to 0.92)	0.69 (0.62 to 0.76)
Former user of at least one product	0.90 (0.85 to 0.95)	0.90 (0.87 to 0.94)

Ban on smoking in outdoor places where social distance cannot be ensured: are you in favour of banning smoking in outdoor places where social distancing cannot be ensured (eg, parks, beaches, entrances of public buildings)?

Ban on e-cigarettes or HTPs in environments where smoking is prohibited: are you in favour of banning the use of e-cigarettes or heated tobacco products in environments where smoking is prohibited?

HTP, heated tobacco product; PR, prevalence ratio.

In our sensitivity analysis, excluding ‘don’t know’ responses (*N* for each outcome variable, as presented in online supplemental table 4), the overall findings remained consistent, with no major variations observed.

## Discussion

This study highlights the significant variation in SHS and SHA exposure across the EU MS. Vulnerable populations, such as younger age groups, those socioeconomically disadvantaged and current or former tobacco and nicotine users, self-reported higher exposure. While the majority of EU residents supported bans on tobacco use in public spaces, support was lower among vulnerable groups, tobacco users and those living without children.

Smoke-free legislation is the foundation of creating smoke-free environments. However, legislation alone is not sufficient; smoke-free policies must be comprehensive and supported by rigorous enforcement to ensure compliance.^21^ This is illustrated by the variation in exposure to SHS from conventional tobacco products in indoor public places, despite the fact that many EU MS have, in principle at least, extensive indoor smoking bans in place. Legislation regarding use of emerging products indoors is not as consistent across EU MS,^22^ which may also partly explain the greater variation in exposure to SHA indoors. Despite issues with enforcement of smoke-free legislation, we found that exposure to SHA, but particularly to SHS, was much lower in indoor spaces compared with all outdoor settings assessed in the survey. This may suggest that indoor bans are effective and that, consequently, the extension to outdoor spaces that has been recommended by the Council of the EU^18^ could lead to similar results outdoors, although reports on use of nicotine and tobacco products in indoor and outdoor spaces might not always be directly comparable.

The gap between the prevalence of exposure in indoor and outdoor spaces also varies between countries, reflecting variation in the prevalence of use but also in existing legislation and in social norms around tobacco and nicotine use.^23^ For instance, in Slovenia, the results reveal a significant disparity, with indoor smoking prevalence being very low (9.4%) compared with much higher rates in outdoor spaces (86.8%). However, outdoor smoking in spaces for children or adolescents is notably lower (56.1%). This discrepancy may be driven by local social norms, where smoking indoors is less socially acceptable or regulated, while outdoor smoking, such as in public spaces or outdoor cafés, may be more culturally tolerated. At the same time, smoking in spaces frequented by children or adolescents is less accepted. These results suggest that, even in the absence of specific outdoor smoking legislation, the cultural shift initiated by indoor bans may extend to outdoor spaces. This highlights the broader impact of comprehensive smoke-free policies, which not only protect public health in public spaces but also encourage healthier behaviours in private settings.^24^ However, further research is needed to better understand the underlying reasons for such discrepancies.

Beyond differences between countries, we also found disparities across various sociodemographic characteristics. These disparities could be explained, to some extent, by the fact that some groups simply frequent certain places more often, thereby increasing their chances of exposure. For example, individuals living with children are more likely to visit outdoor spaces with children and adolescents, such as playgrounds or family-oriented activities, which increases their exposure to SHS or aerosol in these specific environments.^25^ However, some people in highly exposed groups might also prefer places where smoking or vaping is more common, either legally or in violation of the legislation. For instance, current and former tobacco users are more likely to frequent smoke-friendly environments, which leads to higher exposure. Research in Canada supports this, showing that smokers are more likely to report exposure to SHS compared with non-smokers.^26^ Similarly, young people often gather in social settings where their peers hang out, and since e-cigarettes and HTPs are more prevalent among this age group, they become more exposed to SHA.^27 28^ A study in the USA found a similar trend, with reported exposure to SHA among youth increasing alongside the rise in youth e-cigarette use, particularly in environments like schools.^29^ Although our analysis could not disentangle these factors behind reported exposure, it highlights that substantial sections of society are potentially exposed to SHS and SHA and, hence, it is imperative to protect them through appropriate regulations.

Implementing bans, such as those that are included in the EU Council Recommendation, can drastically reduce exposure to SHS and SHA in all settings,^18^ which would minimise these socioeconomic inequalities. According to WHO Europe, though,^30^ public support is critical to ensuring the successful implementation of health policies. Our findings suggest that public attitudes towards bans roughly similar to those included in the EU Council Recommendation also differ across population subgroups in the EU. Countries with higher smoking prevalence, such as Latvia, Belgium and Greece, tend to show lower support for SHA and SHS bans, while Northern European countries with relatively low smoking prevalence show higher support for these outdoor bans.^9^ This study found that former and current users of conventional tobacco and emerging products are less likely to support such bans,^31 32^ likely because these policies directly affect their behaviours. Similarly, countries like Finland, Sweden and the Netherlands, where the prevalence of e-cigarette and HTP use is lower,^9^ also show relatively high support for outdoor bans.

Additionally, individuals living with children were more supportive, likely appreciating the importance of those policies for their children’s health. Similarly, individuals with higher education were more likely to support such policies, possibly because they are more aware of the risks associated with tobacco use.^33^

Overall support for bans of HTP and e-cigarette use was higher than for bans on smoking. However, this may have to do with the wording of the respective questions. Although the question about smoking was explicitly about outdoor spaces, the one regarding HTPs and e-cigarettes mentioned environments where smoking is prohibited, which, at the time of the survey, meant mostly indoor spaces.

### Strengths and limitations

The study draws on a large sample size that includes all 27 current EU MS and employs a consistent set of survey questions. The data are recent and cover exposure to SHA across the EU, specifically in outdoor spaces for the first time, which is an issue of high policy relevance for EU MS. However, some MS have relatively small sample sizes, which might limit the power in subgroup analyses. Furthermore, the reliance on self-reported data introduces potential biases, such as inconsistencies in respondents’ recognition of tobacco use frequency or status. Although interviewers provided verbal descriptions of each tobacco and nicotine product assessed at the beginning of the interview, the absence of accompanying images may have created some confusion. However, this is unlikely to have resulted in substantial misclassification. Additionally, current smoking was assessed through a non-specific question, which respondents may have interpreted in various ways, for example, in terms of frequency of use. Similarly, the question did not explicitly mention waterpipe, so exclusive waterpipe users may have been misclassified as non-smokers. However, exclusive regular waterpipe use was quite rare in the EU in 2023.^9^ It is important to note that the survey questions required participants to report observing tobacco or e-cigarette/HTP use, which we regarded as a proxy of SHS and SHA exposure and does not necessarily reflect personal exposure or quantify the actual level or frequency of exposure. For example, the data does not specify how many individuals were using the quantity used or the frequency of exposure—whether the exposure occurred once in 6 months or daily. As such, we may have underestimated differences between countries or/and socioeconomic groups. We did not account for cultural and contextual differences across countries, which may influence responses and should be considered when interpreting the findings.

## Conclusion

This study highlights significant variations in SHS and SHA exposure across EU MS, showing that younger people and those with more socioeconomic disadvantages are disproportionately affected. To mitigate these disparities, comprehensive legislation and stronger enforcement mechanisms are essential. The 2024 EU Council Recommendation is a bold next step to achieve truly smoke- and aerosol-free environments in the EU. While current and former users of conventional tobacco and emerging products consistently demonstrate lower levels of support for such measures, public support remained high overall. Targeted communication strategies aimed at these groups, alongside the prioritisation of smoke-free measures in areas frequently visited by children and adolescents, could bolster public support for tobacco control policies and provide greater protection for vulnerable populations.

## Supplementary material

10.1136/bmjph-2025-002903 online supplemental table 1

## Data Availability

Data may be obtained from a third party and are not publicly available.
