# Supplementary material for: Exposure to secondhand smoke from cigarettes and secondhand aerosol from tobacco and nicotine products in indoor and outdoor public spaces in the European Union: a cross-sectional study
Source: BMJ Public Health. 2025 Jul 31;3(2):e002903. doi: 10.1136/bmjph-2025-002903 (PMC12314981; doi:10.1136/bmjph-2025-002903)
Supplement: online supplemental table 1 [file bmjph-3-2-s001.docx]

**Supplementary Table 1. Descriptive demographics**

| Variables | *N* | Weighted Proportion |
| --- | --- | --- |
| **Gender** | | |
| Male | 12,179 | 48.5% |
| Female | 14,122 | 51.5% |
| Missing | 57 |  |
| **Age (years)** | | |
| 15-24 | 2,386 | 12.3% |
| 25-39 | 5,170 | 22.1% |
| 40-54 | 6,577 | 25.1% |
| 55+ | 12,222 | 40.1% |
| Missing | 3 |  |
| **Difficulty paying bills** | | |
| Almost never/never | 17,328 | 65.8% |
| From time to time/most of the time | 8,846 | 34.2% |
| Missing | 184 |  |
| **Community type** | | |
| Rural | 8,972 | 31.4% |
| Urban | 17,377 | 68.6% |
| Missing | 9 |  |
| **Education (age at completion)** | | |
| 0-15 years | 2,968 | 13.5% |
| 16-19 years | 11,188 | 43.5% |
| 20+ years | 9,858 | 33.3% |
| Still studying | 1,972 | 9.7% |
| Missing | 372 |  |
| **Living with children** | | |
| Yes | 8,621 | 32.9% |
| No | 17,570 | 67.2% |
| Missing | 167 |  |
| **Employment status** | | |
| Employed | 14,366 | 55.9% |
| Unemployed | 976 | 4.3% |
| Students/house persons/retired | 11,016 | 39.9% |
| Missing | 0 |  |
| **Current Smoking Status** | | |
| Never Smoker | 14,322 | 55.7% |
| Current Smoker | 6,317 | 24.6% |
| Former Smoker | 5,683 | 19.7% |
| Missing | 36 |  |
| **Current Status of E-Cigarette and HTP Use** | | |
| Never Used E-cigarettes or HTPs | 21,882 | 84.0% |
| Current User of at Least One Product | 1,161 | 4.6% |
| Former User of at Least One Product | 2,915 | 11.5% |
| Missing | 400 |  |
| **In the last six months, were people smoking in indoor public spaces** | | |
| Yes | 5,480 | 21.5% |
| No/not visited/don’t know | 20,878 | 78.5% |
| Missing | 0 |  |
| **In the last six months, were people smoking on an outdoor terrace** | | |
| Yes | 16,947 | 68.8% |
| No/not visited/don’t know | 9,411 | 31.2% |
| Missing | 0 |  |
| **In the last six months, were people smoking in outdoor spaces for children or adolescents** | | |
| Yes | 9,481 | 37.5% |
| No/not visited/don’t know | 16,877 | 62.5% |
| Missing | 0 |  |
| **In the last six months, were people smoking at outdoor events** | | |
| Yes | 16,277 | 64.0% |
| No/not visited/don’t know | 10,081 | 36.0% |
| Missing | 0 |  |
| **In the last six months, were people smoking in outdoor public spaces** | | |
| Yes | 18,924 | 72.6% |
| No/not visited/don’t know | 7,434 | 27.4% |
| Missing | 0 |  |
| **In the last six months, were people smoking at open-air public transportation stations** | | |
| Yes | 17,500 | 65.8% |
| No/not visited/don’t know | 8,858 | 34.2% |
| Missing | 0 |  |
| **In the last six months, were people using e-cigarettes or heated tobacco products in indoor public spaces** | | |
| Yes | 8,856 | 35.8% |
| No/not visited/don’t know | 17,502 | 64.2% |
| Missing | 0 |  |
| **In the last six months, were people using e-cigarettes or heated tobacco products on an outdoor terrace** | | |
| Yes | 15,831 | 63.2% |
| No/not visited/don’t know | 10,527 | 36.8% |
| Missing |  |  |
| **In the last six months, were people using e-cigarettes or heated tobacco products in outdoor spaces for children or adolescents** | | |
| Yes | 10,594 | 41.5% |
| No/not visited/don’t know | 15,764 | 58.6% |
| Missing | 0 |  |
| **In the last six months, were people using e-cigarettes or heated tobacco products at outdoor events** | | |
| Yes | 15,712 | 60.9% |
| No/not visited/don’t know | 10,646 | 39.1% |
| Missing | 0 |  |
| **In the last six months, were people using e-cigarettes or heated tobacco products in outdoor public spaces** | | |
| Yes | 17,105 | 65.2% |
| No/not visited/don’t know | 9,253 | 34.8% |
| Missing | 0 |  |
| **In the last six months, were people using e-cigarettes or heated tobacco products at open-air public transportation stations** | | |
| Yes | 16,281 | 61.6% |
| No/not visited/don’t know | 10,077 | 38.4% |
| Missing | 0 |  |
| **Ban on smoking in outdoor places where social distance cannot be ensured** | | |
| In favour | 15,681 | 56.5% |
| Not in favour/don’t know | 10,677 | 43.5% |
| Missing | 0 |  |
| **Ban on e-cigarettes or heated tobacco products in environments where smoking is prohibited** | | |
| In favour | 17,931 | 65.8% |
| Not in favour/don’t know | 8,427 | 34.2% |
| Missing | 0 |  |
| ***N*** | 26,358 |  |

Ban on Smoking in Outdoor Places Where Social Distance Cannot Be Ensured: Are you in favour of banning smoking in outdoor places where social distancing cannot be ensured (e.g., parks, beaches, entrances of public buildings)?

Ban on E-cigarettes or HTPs in Environments Where Smoking is Prohibited: Are you in favour of banning the use of e-cigarettes or heated tobacco products in environments where smoking is prohibited?

**Supplementary Table 2. Exposure to traditional tobacco smoke in the past six months across 27 EU Member States (*N* = 26,358)**

| Country | In the last six months, were people smoking… | | | | | |
| --- | --- | --- | --- | --- | --- | --- |
|  | In Indoor Public Spaces | On Outdoor Terraces | In Outdoor Spaces for Children or Adolescents | At Outdoor Events | In Outdoor Public Spaces | At Open-Air Public Transportation Stations |
|  | Weighted % with 95% CI | | | | | |
| Austria | 19.1%  (16.6%- 21.8%) | 66.8%  (63.5%- 70.0%) | 35.0%  (31.8%- 38.3%) | 52.2%  (48.8%- 55.6%) | 62.7%  (59.4%- 65.9%) | 57.2%  (53.8%- 60.5%) |
| Belgium | 24.1%  (21.3%- 27.2%) | 75.0%  (71.7%- 78.0%) | 33.7%  (30.5%- 37.1%) | 67.1%  (63.6%- 70.4%) | 72.8%  (69.4%- 75.9%) | 62.6%  (59.0%- 66.0%) |
| Bulgaria | 46.3%  (43.2%- 49.4%) | 74.8%  (72.0%- 77.4%) | 64.0%  (60.9%- 66.9%) | 64.0%  (60.9%- 66.9%) | 77.3%  (74.6%- 79.8%) | 79.6%  (77.0%- 82.0%) |
| Croatia | 40.5%  (37.2%- 43.8%) | 73.5%  (70.3%- 76.4%) | 64.2%  (60.9%- 67.4%) | 79.9%  (77.0%- 82.5%) | 85.8%  (83.2%- 88.0%) | 84.1%  (81.5%- 86.5%) |
| Cyprus Republic | 46.2%  (41.5%- 51.1%) | 83.4%  (79.6%- 86.7%) | 58.5%  (53.6%- 63.2%) | 80.9%  (76.8%- 84.4%) | 79.0%  (74.9%- 82.7%) | 71.2%  (66.6%- 75.4%) |
| Czech Republic | 12.5%  (10.4%- 15.0%) | 58.3%  (54.7%- 61.8%) | 24.3%  (21.4%- 27.4%) | 62.1%  (58.5%- 65.5%) | 59.4%  (55.8%- 62.9%) | 55.7%  (52.2%- 59.3%) |
| Denmark | 21.0%  (18.1%- 24.1%) | 72.7%  (69.5%- 75.7%) | 28.2%  (24.9%- 31.6%) | 68.6%  (65.4%- 71.7%) | 76.7%  (73.7%- 79.3%) | 62.7%  (59.3%- 66.0%) |
| Estonia | 14.9%  (12.8%- 17.3%) | 57.6%  (54.4%- 60.7%) | 28.2%  (25.5%- 31.1%) | 58.0%  (54.8%- 61.0%) | 78.6%  (75.8%- 81.0%) | 78.8%  (76.1%- 81.2%) |
| Finland | 8.3%  (6.4%- 10.8%) | 68.9%  (65.5%- 72.1%) | 21.9%  (18.9%- 25.3%) | 62.5%  (58.9%- 65.9%) | 75.2%  (72.1%- 78.0%) | 76.8%  (73.8%- 79.5%) |
| France | 25.9%  (23.0%- 29.0%) | 82.1%  (79.4%- 84.5%) | 36.1%  (32.8%- 39.5%) | 69.2%  (66.0%- 72.3%) | 79.4%  (76.6%- 82.0%) | 72.3%  (69.2%- 75.3%) |
| Germany | 17.4%  (15.3%- 19.6%) | 70.3%  (67.7%- 72.8%) | 30.3%  (27.8%- 32.9%) | 65.4%  (62.7%- 68.0%) | 71.5%  (68.9%- 73.9%) | 73.0%  (70.4%- 75.4%) |
| Greece | 26.2%  (23.6%- 29.1%) | 87.0%  (84.7%- 89.0%) | 55.3%  (52.2%- 58.4%) | 88.5%  (86.3%- 90.3%) | 93.9%  (92.3%- 95.3%) | 83.8%  (81.3%- 86.0%) |
| Hungary | 8.9%  (7.2%- 11.0%) | 29.5%  (26.7%- 32.5%) | 14.4%  (12.3%- 16.8%) | 39.9%  (36.7%- 43.1%) | 48.6%  (45.4%- 51.8%) | 40.0%  (36.9%- 43.2%) |
| Ireland | 14.8%  (12.5%- 17.4%) | 70.5%  (67.4%- 73.4%) | 43.8%  (40.4%- 47.2%) | 60.5%  (57.2%- 63.7%) | 73.8%  (70.9%- 76.6%) | 56.5%  (53.1%- 59.7%) |
| Italy | 26.2%  (23.6%- 29.1%) | 68.4%  (65.4%- 71.2%) | 52.5%  (49.3%- 55.6%) | 64.9%  (61.8%- 67.8%) | 69.9%  (66.9%- 72.7%) | 63.8%  (60.8%- 66.8%) |
| Latvia | 15.4%  (13.2%- 17.9%) | 31.6%  (28.6%- 34.7%) | 15.5%  (13.3%- 18.1%) | 39.0%  (35.8%- 42.2%) | 53.0%  (49.7%- 56.2%) | 53.5%  (50.2%- 56.7%) |
| Lithuania | 17.1%  (14.5%- 20.0%) | 34.7%  (31.4%- 38.1%) | 33.6%  (30.3%- 36.9%) | 53.0%  (49.6%- 56.4%) | 64.4%  (61.1%- 67.5%) | 58.8%  (55.4%- 62.1%) |
| Luxembourg | 21.1%  (17.4%- 25.3%) | 80.7%  (76.8%- 84.0%) | 36.2%  (31.7%- 41.0%) | 68.4%  (63.8%- 72.6%) | 81.1%  (77.1%- 84.5%) | 86.3%  (82.9%- 89.1%) |
| Malta | 37.9%  (32.9%- 43.1%) | 91.9%  (88.7%- 94.2%) | 50.5%  (45.2%- 55.7%) | 73.0%  (68.3%- 77.3%) | 89.2%  (85.9%- 91.8%) | 81.2%  (76.9%- 84.8%) |
| Netherlands | 27.5%  (24.3%- 31.0%) | 87.2%  (84.6%- 89.5%) | 32.3%  (29.0%- 35.9%) | 76.7%  (73.6%- 79.5%) | 84.7%  (82.0%- 87.1%) | 70.2%  (66.9%- 73.3%) |
| Poland | 20.9%  (18.1%- 24.1%) | 41.3%  (37.9%- 44.8%) | 38.8%  (35.4%- 42.3%) | 49.4%  (45.9%- 52.8%) | 58.7%  (55.3%- 62.0%) | 54.4%  (50.9%- 57.7%) |
| Portugal | 17.1%  (14.8%- 19.6%) | 81.7%  (79.2%- 84.0%) | 35.5%  (32.5%- 38.6%) | 75.2%  (72.5%- 77.8%) | 78.7%  (76.1%- 81.2%) | 74.4%  (71.6%- 77.0%) |
| Romania | 13.6%  (11.6%- 15.9%) | 52.7%  (49.6%- 55.8%) | 30.2%  (27.4%- 33.1%) | 51.6%  (48.5%- 54.7%) | 62.9%  (59.8%- 65.9%) | 58.0%  (54.9%- 61.0%) |
| Slovakia | 23.8%  (21.0%- 26.8%) | 48.3%  (44.9%- 51.7%) | 23.2%  (20.4%- 26.3%) | 53.6%  (50.2%- 57.0%) | 61.9%  (58.5%- 65.1%) | 71.2%  (68.0%- 74.1%) |
| Slovenia | 9.4%  (7.6%- 11.5%) | 86.8%  (84.5%- 88.8%) | 56.1%  (52.8%- 59.4%) | 79.8%  (77.2%- 82.3%) | 85.6%  (83.2%- 87.7%) | 84.6%  (82.0%- 86.8%) |
| Spain | 21.3%  (18.8%- 24.0%) | 81.3%  (78.8%- 83.6%) | 40.9%  (37.8%- 43.9%) | 66.8%  (63.8%- 69.6%) | 78.9%  (76.3%- 81.3%) | 53.7%  (50.7%- 56.8%) |
| Sweden | 15.7%  (12.9%- 18.9%) | 36.7%  (32.8%- 40.7%) | 32.1%  (28.3%- 36.1%) | 52.2%  (48.2%- 56.1%) | 82.6%  (79.7%- 85.1%) | 76.7%  (73.4%- 79.7%) |
| EU | 21.5%  (20.7%- 22.4%) | 68.8%  (67.9%- 69.7%) | 37.5%  (36.5%-38.4%) | 64.0%  (63.0%-64.9%) | 72.6%  (71.7%-73.4%) | 65.8%  (64.8%-66.7%) |

* Abbreviations: CI = Confidence Interval

Indoor Public Spaces: Indoor public spaces where people normally do not smoke (e.g. restaurants, bars, shopping malls, airports, concert halls)

Outdoor Terraces: Outdoor terraces of a drinking or eating establishment

Outdoor Spaces: Outdoor spaces intended for use by children or adolescents (e.g. nursery and school courtyard, playgrounds)

Outdoor Events: Outdoor events (e.g. open-air concerts, sport matches, markets)

Outdoor Public Spaces: Outdoor public spaces (e.g., park, beach, entrance to public buildings)

Open-Air Public Transportation Stations: Open-air public transportation stations (e.g. bus, tram or train stations)

**Supplementary Table 3. Exposure to e-cigarettes and heated tobacco products in the past six months across 27 EU Member States (*N* = 26,358)**

| Country | In the last six months, were people using e-cigarettes or heated tobacco products… | | | | | |
| --- | --- | --- | --- | --- | --- | --- |
|  | In Indoor Public Spaces | On Outdoor Terraces | In Outdoor Spaces for Children or Adolescents | At Outdoor Events | In Outdoor Public Spaces | At Open-Air Public Transportation Stations |
|  | Weighted % with 95% CI | | | | | |
| Austria | 27.8%  (24.8%- 31.0%) | 56.9%  (53.5%- 60.2%) | 35.3%  (32.1%- 38.6%) | 45.9%  (42.5%- 49.3%) | 52.5%  (49.1%- 55.9%) | 48.7%  (45.3%- 52.1%) |
| Belgium | 37.0%  (33.6%- 40.5%) | 70.5%  (67.1%- 73.7%) | 44.1%  (40.6%- 47.7%) | 65.8%  (62.3%- 69.1%) | 68.8%  (65.3%- 72.0%) | 64.8%  (61.2%- 68.1%) |
| Bulgaria | 58.1%  (54.9%- 61.1%) | 64.2%  (61.1%- 67.1%) | 59.1%  (56.0%- 62.1%) | 62.0%  (58.9%- 65.0%) | 64.4%  (61.4%- 67.4%) | 65.4%  (62.4%- 68.3%) |
| Croatia | 52.1%  (48.7%- 55.5%) | 70.2%  (66.9%- 73.2%) | 62.8%  (59.4%- 66.0%) | 74.2%  (71.1%- 77.1%) | 77.3%  (74.4%- 80.1%) | 75.6%  (72.6%- 78.4%) |
| Cyprus Republic | 53.7%  (48.9%- 58.5%) | 76.0%  (71.7%- 79.9%) | 61.1%  (56.3%- 65.7%) | 74.9%  (70.5%- 78.9%) | 71.7%  (67.2%- 75.9%) | 68.4%  (63.7%- 72.7%) |
| Czech Republic | 43.7%  (40.2%- 47.2%) | 65.2%  (61.7%- 68.5%) | 39.5%  (36.1%- 43.0%) | 68.6%  (65.1%- 71.8%) | 65.9%  (62.3%- 69.2%) | 64.5%  (61.1%- 67.9%) |
| Denmark | 34.5%  (31.0%- 38.1%) | 63.4%  (60.0%- 66.7%) | 34.2%  (30.7%- 37.8%) | 62.3%  (58.8%- 65.6%) | 67.2%  (63.9%- 70.4%) | 60.3%  (56.8%- 63.6%) |
| Estonia | 37.5%  (34.6%- 40.6%) | 69.3%  (66.3%- 72.1%) | 43.6%  (40.5%- 46.8%) | 70.9%  (67.9%- 73.7%) | 79.9%  (77.2%- 82.4%) | 77.9%  (75.1%- 80.4%) |
| Finland | 21.9%  (18.7%- 25.3%) | 50.8%  (47.1%- 54.5%) | 21.6%  (18.6%- 25.1%) | 48.9%  (45.2%- 52.6%) | 53.0%  (49.3%- 56.7%) | 55.9%  (52.2%- 59.5%) |
| France | 48.6%  (45.2%- 52.1%) | 80.4%  (77.5%- 83.0%) | 47.8%  (44.3%- 51.2%) | 72.6%  (69.5%- 75.5%) | 77.5%  (74.5%- 80.2%) | 74.8%  (71.8%- 77.6%) |
| Germany | 25.7%  (23.3%- 28.2%) | 57.5%  (54.7%- 60.2%) | 32.1%  (29.6%- 34.8%) | 56.0%  (53.2%- 58.7%) | 59.8%  (57.1%- 62.6%) | 60.3%  (57.5%- 63.0%) |
| Greece | 37.8%  (34.9%- 40.9%) | 86.5%  (84.3%- 88.5%) | 61.6%  (58.5%- 64.6%) | 89.4%  (87.3%- 91.2%) | 92.8%  (91.0%- 94.3%) | 86.9%  (84.6%- 88.9%) |
| Hungary | 14.0%  (11.9%- 16.3%) | 30.8%  (27.9%- 33.8%) | 16.0%  (13.7%- 18.5%) | 38.9%  (35.8%- 42.1%) | 44.1%  (40.9%- 47.3%) | 41.3%  (38.2%- 44.5%) |
| Ireland | 42.6%  (39.2%- 45.9%) | 72.5%  (69.5%- 75.3%) | 56.9%  (53.6%- 60.2%) | 67.3%  (64.2%- 70.3%) | 74.8%  (71.9%- 77.5%) | 63.9%  (60.6%- 67.0%) |
| Italy | 48.3%  (45.2%- 51.4%) | 69.1%  (66.1%- 71.9%) | 56.8%  (53.7%- 59.9%) | 68.3%  (65.3%- 71.1%) | 69.0%  (66.0%- 71.8%) | 67.1%  (64.0%- 69.9%) |
| Latvia | 21.5%  (18.9%- 24.4%) | 34.5%  (31.4%- 37.8%) | 20.1%  (17.5%- 22.9%) | 37.7%  (34.6%- 41.0%) | 48.2%  (45.0%- 51.5%) | 48.2%  (45.0%- 51.5%) |
| Lithuania | 27.5%  (24.4%- 30.8%) | 41.7%  (38.3%- 45.1%) | 44.7%  (41.3%- 48.2%) | 58.2%  (54.9%- 61.5%) | 65.5%  (62.3%- 68.6%) | 60.5%  (57.2%- 63.7%) |
| Luxembourg | 27.2%  (23.1%- 31.9%) | 76.5%  (72.1%- 80.3%) | 43.5%  (38.7%- 48.4%) | 70.4%  (65.9%- 74.6%) | 77.3%  (73.0%- 81.1%) | 80.9%  (77.0%- 84.3%) |
| Malta | 53.4%  (48.2%- 58.6%) | 83.0%  (79.0%- 86.5%) | 51.8%  (46.5%- 57.0%) | 68.8%  (63.9%- 73.2%) | 82.7%  (78.8%- 86.0%) | 79.1%  (74.7%- 82.9%) |
| Netherlands | 41.7%  (38.1%- 45.4%) | 71.5%  (68.2%- 74.7%) | 35.9%  (32.4%- 39.5%) | 66.1%  (62.7%- 69.4%) | 71.4%  (68.1%- 74.5%) | 61.7%  (58.2%- 65.0%) |
| Poland | 27.4%  (24.3%- 30.7%) | 46.1%  (42.6%- 49.6%) | 44.4%  (40.9%- 47.9%) | 51.3%  (47.8%- 54.7%) | 56.4%  (53.0%- 59.8%) | 55.2%  (51.7%- 58.5%) |
| Portugal | 24.5%  (21.8%- 27.3%) | 74.1%  (71.3%- 76.7%) | 34.0%  (31.0%- 37.0%) | 69.8%  (66.9%- 72.6%) | 72.8%  (70.0%- 75.5%) | 68.8%  (65.9%- 71.6%) |
| Romania | 36.2%  (33.3%- 39.2%) | 53.1%  (50.0%- 56.2%) | 39.5%  (36.5%- 42.5%) | 53.8%  (50.6%- 56.9%) | 57.7%  (54.5%- 60.8%) | 56.0%  (52.8%- 59.1%) |
| Slovakia | 29.1%  (26.1%- 32.3%) | 49.2%  (45.8%- 52.6%) | 27.3%  (24.4%- 30.5%) | 56.4%  (53.0%- 59.7%) | 60.4%  (57.1%- 63.7%) | 67.8%  (64.6%- 70.8%) |
| Slovenia | 29.6%  (26.5%- 32.8%) | 81.8%  (79.2%- 84.2%) | 62.5%  (59.2%- 65.7%) | 79.1%  (76.3%- 81.5%) | 81.6%  (79.1%- 84.0%) | 80.0%  (77.3%- 82.5%) |
| Spain | 28.9%  (26.1%- 31.8%) | 61.7%  (58.7%- 64.7%) | 33.8%  (30.9%- 36.8%) | 51.6%  (48.5%- 54.7%) | 58.0%  (54.9%- 61.0%) | 42.2%  (39.2%- 45.3%) |
| Sweden | 32.8%  (28.9%- 36.9%) | 38.0%  (34.0%- 42.0%) | 31.0%  (27.1%- 35.1%) | 44.6%  (40.6%- 48.6%) | 61.9%  (58.1%- 65.5%) | 55.4%  (51.4%- 59.2%) |
| EU | 35.8%  (34.8%- 36.7%) | 63.2%  (62.2%- 64.1%) | 41.5%  (40.5%- 42.4%) | 60.9%  (59.9%- 61.9%) | 65.2%  (64.2%- 66.1%) | 61.6%  (60.6%- 62.6%) |

* Abbreviations: CI = Confidence Interval; Heated Tobacco Product = HTP

Indoor Public Spaces: Indoor public spaces where people normally do not smoke (e.g. restaurants, bars, shopping malls, airports, concert halls)

Outdoor Terraces: Outdoor terraces of a drinking or eating establishment

Outdoor Spaces: Outdoor spaces intended for use by children or adolescents (e.g. nursery and school courtyard, playgrounds)

Outdoor Events: Outdoor events (e.g. open-air concerts, sport matches, markets)

Outdoor Public Spaces: Outdoor public spaces (e.g., park, beach, entrance to public buildings)

Open-Air Public Transportation Stations: Open-air public transportation stations (e.g. bus, tram or train stations)

**Supplementary Table 4. Distribution of “don’t know” responses across outcome variables (*N* = 26,358)**

| Variables | *N* | Weighted Proportion |
| --- | --- | --- |
| **In the last six months, were people smoking in indoor public spaces** | | |
| Don’t know | 1,405 | 5.2% |
| **In the last six months, were people smoking on an outdoor terrace** | | |
| Don’t know | 1,377 | 4.9% |
| **In the last six months, were people smoking in outdoor spaces for children or adolescents** | | |
| Don’t know | 1,966 | 7.1% |
| **In the last six months, were people smoking at outdoor events** | | |
| Don’t know | 1,944 | 7.1% |
| **In the last six months, were people smoking in outdoor public spaces** | | |
| Don’t know | 1,352 | 4.9% |
| **In the last six months, were people smoking at open-air public transportation stations** | | |
| Don’t know | 1,557 | 5.8% |
| **In the last six months, were people using e-cigarettes or heated tobacco products in indoor public spaces** | | |
| Don’t know | 2,841 | 10.1% |
| **In the last six months, were people using e-cigarettes or heated tobacco products on an outdoor terrace** | | |
| Don’t know | 2,615 | 9.4% |
| **In the last six months, were people using e-cigarettes or heated tobacco products in outdoor spaces for children or adolescents** | | |
| Don’t know | 3,174 | 11.3% |
| **In the last six months, were people using e-cigarettes or heated tobacco products at outdoor events** | | |
| Don’t know | 2,969 | 10.6% |
| **In the last six months, were people using e-cigarettes or heated tobacco products in outdoor public spaces** | | |
| Don’t know | 2,666 | 9.5% |
| **In the last six months, were people using e-cigarettes or heated tobacco products at open-air public transportation stations** | | |
| Don’t know | 2,766 | 10.0% |
| **Ban on smoking in outdoor places where social distance cannot be ensured** | | |
| Don’t know | 1,949 | 7.6% |
| **Ban on e-cigarettes or heated tobacco products in environments where smoking is prohibited** | | |
| Don’t know | 2,089 | 8.0% |

Ban on Smoking in Outdoor Places Where Social Distance Cannot Be Ensured: Are you in favour of banning smoking in outdoor places where social distancing cannot be ensured (e.g., parks, beaches, entrances of public buildings)?

Ban on E-cigarettes or HTPs in Environments Where Smoking is Prohibited: Are you in favour of banning the use of e-cigarettes or heated tobacco products in environments where smoking is prohibited?
